# Herzinsuffizienzprotektion bei Patienten mit Diabetes mellitus Typ 2 durch SGLT2-Inhibitoren – Evidenzlage und mögliche Mechanismen: Systematische Übersicht

**DOI:** 10.1007/s00059-020-04994-0

**Published:** 2020-10-12

**Authors:** Andreas J. Rieth, Christian W. Hamm, Christoph Wanner, Veselin Mitrovic, Till Keller

**Affiliations:** 1 Kerckhoff-Klinik, Abteilung für Kardiologie, Campus Kerckhoff der Justus-Liebig-Universität Gießen, Benekestr. 2–8, 61231 Bad Nauheim, Deutschland; 2 Standort Rhein-Main, Deutsches Zentrum für Herz-Kreislauf-Forschung, Frankfurt am Main, Deutschland; 3 Medizinische Klinik I, Kardiologie, Justus-Liebig-Universität Gießen, Gießen, Deutschland; 4 Abteilung Nephrologie, Universitätsklinikum Würzburg, Würzburg, Deutschland

**Keywords:** Empagliflozin, Canagliflozin, Dapagliflozin, Kardioprotektiver Effekt, Kardiorenale Interaktion, Empagliflozin, Canagliflozin, Dapagliflozin, Cardioprotective effect, Cardiorenal interaction

## Abstract

**Hintergrund:**

Hemmstoffe des renalen Natrium-Glukose-Kotransporters 2 („sodium glucose-linked transporter 2“; SGLT2i) scheinen außer der antidiabetischen auch eine kardioprotektive Wirkung zu besitzen; deren Mechanismus ist jedoch unklar.

**Methoden:**

Selektive Literaturrecherche in PubMed (Fokus Herzinsuffizienz und Wirkmechanismen).

**Ergebnisse:**

Unter Therapie mit 3 der untersuchten Substanzen kam es im Vergleich mit Placebo zu weniger herzinsuffizienzbedingten Krankenhausaufenthalten, allerdings mit einer relativ hohen Anzahl von Behandlungen pro verhindertes Ereignis (72–117). Außer einer stärkeren Gewichtsabnahme und Blutdrucksenkung unter dem Verum gegenüber Placebo fiel eine Zunahme des kardioprotektiven Effekts bei stärker eingeschränkter Nierenfunktion auf.

**Schlussfolgerung:**

Ein moderater herzinsuffizienzpräventiver Effekt von Hemmstoffen des renalen SGLT2 kann bei Diabetikern als gesichert gelten. Ein wesentlicher Wirkmechanismus beruht wahrscheinlich auf einem nephroprotektiven Effekt mit Modulation der kardiorenalen Interaktion, was jedoch weiterer Abklärung bedarf.

## Abstract (en)

**Background:**

Renal sodium-glucose cotransporter‑2 (SGLT2) inhibitors seem to have a cardioprotective effect beyond the antidiabetic effect. The underlying mechanisms are unclear.

**Methods:**

Selective search in PubMed with a focus on heart failure endpoints and possible mechanisms of action.

**Results:**

During treatment with three of the substances analyzed, there were fewer hospitalizations for heart failure compared with placebo; however, the numbers needed to treat within the primary analyses were relatively high (72–117). We found that loss of weight and lowering of blood pressure were more pronounced during treatment with verum than with placebo and an association of the preventive effect with more severely impaired renal function.

**Conclusion:**

The SGLT2 inhibitors show a moderate heart failure protective effect in diabetic patients. It is likely that a nephroprotective effect with modulation of the cardiorenal interaction is an important part of the mechanism of action but this must be substantiated in further investigations.

## Hintergrund

Herzinsuffizienz (HI) ist eine der gefürchtetsten Folgeerkrankungen des Diabetes mellitus mit ungünstiger Prognose und erhöhtem Hospitalisationsrisiko. Medikamente zur Behandlung des Diabetes mellitus können einen günstigen, neutralen oder ungünstigen Einfluss auf eine HI ausüben [1]. Die HI-Leitlinien der European Society of Cardiology (ESC) von 2016 enthalten nur wenige Empfehlungen zur antidiabetischen Therapie bei HI: eine Kontraindikation (Klasse III, Evidenzlevel A: Daten aus mehreren randomisierten Studien oder Metaanalysen) für Thiazolidindione und eine IIa-C-Empfehlung (IIa: „sollte erwogen werden“; Evidenzlevel C: Expertenmeinung) für Metformin. Zur HI-Prophylaxe und Prognoseverbesserung wird an anderer Stelle Empagliflozin, ein blutzuckersenkender Inhibitor des Natrium-Glukose-Kotransporters 2 („sodium glucose-linked transporter 2“; SGLT2i), empfohlen (Klasse IIa, Evidenzlevel B: Daten aus einer einzelnen randomisierten Studie oder großen nichtrandomisierten Studien; [2]). Diese Wirkstoffklasse erfährt derzeit großes Interesse mit einer Vielzahl aktueller Publikationen. Während ein Klasseneffekt der SGLT2i mit positiven Auswirkungen auf eine HI von vielen Autoren angenommen wird, ist der Stellenwert beteiligter Mechanismen (u. a. Blutdruck- und Gewichtsabnahme, Natrium- und Flüssigkeitsverlust, verlangsamter Nierenfunktionsverlust, reduzierter oxidativer Stress, verbesserter Metabolismus) unklar [3].

Die vorliegende Arbeit hat sich daher zum Ziel gesetzt, folgenden Fragen nachzugehen:

- Erlauben die vorliegenden Studiendaten Rückschlüsse über im Kontext der HI-Pathophysiologie plausible zugrunde liegende Mechanismen?
- Zeigt die vorhandene Evidenz aus randomisierten, kontrollierten Studien eine Verhinderung von HI-Ereignissen durch SGLT2i als Klasseneffekt?
- Wie ist das Nutzen-Risiko-Verhältnis durch unerwünschte Arzneimittelwirkungen zu bewerten?

## Methoden

Die vorliegende Übersichtsarbeit wurde vorab in PROSPERO (International Prospective Register of Systematic Reviews) registriert (No. CRD42019122656) und nach den PRISMA(Preferred Reporting Items for Systematic Reviews and Meta-Analyses)-Empfehlungen [4] erstellt. In einer selektiven PubMed-Datenbankrecherche fanden folgende Suchbegriffe Verwendung: Date – Publication: 2009/02/09 to 2019/02/09 AND Language: English AND Title/Abstract: Heart Failure AND Title/Abstract: Cardiovascular AND Title/Abstract: Sodium Glucose Cotransporter 2 Inhibitor AND All Fields: Controlled Trial. Alle Originalarbeiten, die über HI-Ereignisse oder Biomarker mit Bezug zur HI als primären, sekundären oder Sicherheitsendpunkt berichteten oder die eine Subgruppenanalyse unter Einbezug von HI durchgeführt hatten, wurden einer näheren Auswertung unterzogen. Berücksichtigung fanden randomisierte klinische Studien, Beobachtungsstudien und Metaanalysen, sofern weitere außer den bereits einbezogenen Studien analysiert wurden oder wesentliche neue Aspekte zum Vorschein kamen. Bei dem erstellten Datenerfassungsbogen standen Daten zur absoluten Risikoreduktion und zur Anzahl von Behandlungen pro verhindertes Ereignis („number needed to treat“, NNT) im Zentrum. Das Bias-Risiko wurde unter Nutzung des Cochrane-RoB-2-Tools [5] erfasst, und es wurden nur randomisierte Studien mit einem niedrigen Bias-Risiko eingeschlossen.

## Ergebnisse

Unter den 122 Suchergebnissen fanden sich 9 randomisierte Studien, 10 Post-hoc-Analysen, 2 retrospektive Beobachtungsstudien, 32 Metaanalysen, 12 Studienprotokolle/Vorstellungen des Studienaufbaus, 54 Übersichtsartikel und 3 Leitlinien/Positionspapiere.

**Figure 1:**
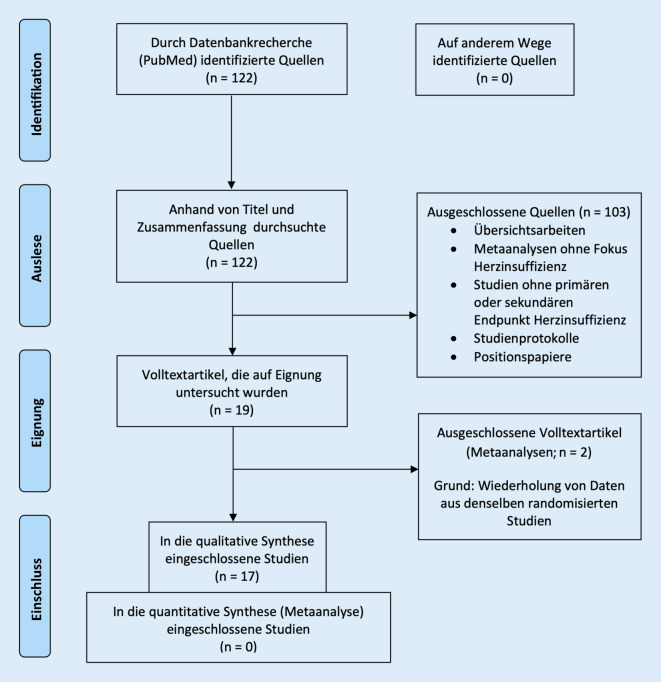

Von einer weiteren Analyse ausgeschlossen wurden Übersichtsartikel, Leitlinien/Positionspapiere und Vorstellungen des Studienaufbaus; weiterhin alle Studien ohne HI-Endpunkte und Metaanalysen ohne Fokus auf HI. Ferner wurden 2 Metaanalysen ausgeschlossen, deren Datenbasis bereits Grundlage anderer eingeschlossener Analysen war (Abb. 1). So kamen letztlich 4 randomisierte Studien, 2 Beobachtungsstudien, 1 Post-hoc-Analyse und 10 Metaanalysen zur Auswertung.

Die größten randomisierten Studien EMPA-REG OUTCOME (Empagliflozin Cardiovascular Outcome Event Trial in Type 2 Diabetes Mellitus Patients; Prüfsubstanz: Empagliflozin), CANVAS (Canagliflozin Cardiovascular Assessment Study; Canagliflozin) und DECLARE-TIMI 58 (Dapagliflozin Effect on Cardiovascular Events – Thrombolysis in Myocardial Infarction 58; Dapagliflozin) prägten alle evaluierten Metaanalysen entscheidend.

### Randomisierte klinische Studien

Für die 3 SGLT2i-Wirkstoffe aus den oben genannten randomisierten Studien steht mit insgesamt über 34.000 Studienteilnehmern eine umfangreiche Datenbasis zur Verfügung.

**Figure 2:**
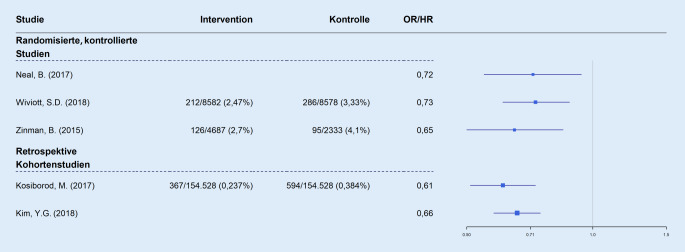

In EMPA-REG OUTCOME (2015) wurden erstmals kardiovaskulär bedingter Tod und Hospitalisation wegen HI unter einer Therapie mit Empagliflozin im Vergleich zu Placebo bei 7020 Patienten mit Typ-2-Diabetes mellitus untersucht [6]. Eine HI hatten anamnestisch 9,90 % der Patienten in der Verum- und 10,50 % in der Placebogruppe. Eine geschätzte glomeruläre Filtrationsrate (eGFR) von weniger als 30 ml/min/1,73 m^2^ war Ausschlusskriterium; eine eGFR von weniger als 60 ml/min/1,73 m^2^ hatten aber 26 % der Studienteilnehmer, sodass hier unter allen randomisierten Studien der Anteil niereninsuffizienter Patienten am größten war. Neben einem positiven Effekt auf den kombinierten primären Endpunkt kardiovaskuläre Sterblichkeit/Myokardinfarkt/Schlaganfall zeigte sich auch ein protektiver Effekt von Empagliflozin auf den sekundären Endpunkt HI-bedingte Hospitalisation. Bei insgesamt relativ geringer Häufigkeit waren Hospitalisationen wegen HI unter Empagliflozintherapie signifikant seltener als unter Placebo (2,70 vs. 4,10 %, absolute Risikoreduktion: 1,40; NNT: 72; relatives Risiko [HR]: 0,65 [95 %- KI: 0,50–0,85]; *p* = 0,002). Mit diesem (fokussiert dargestellten) Ergebnis hatte erstmals ein orales Antidiabetikum eine Verbesserung „harter“ kardiovaskulärer Endpunkte gezeigt. Begleiteffekte ohne Signifikanzberechnung in EMPA-REG OUTCOME waren eine stärkere Gewichtsabnahme (−2,9 vs. −1,8 kg) und eine Reduktion des systolischen Blutdrucks (−2,0 vs. 0 mm Hg) unter Empagliflozin 25 mg gegenüber Placebo. Unerwünschte Arzneimittelwirkungen traten in beiden Gruppen insgesamt vergleichbar häufig auf (Empagliflozin: 90 [20 %], Placebo: 91 [70 %]). Die Rate an Genitalinfektionen war dabei unter Empagliflozin höher (6,40 vs. 1,80 %; *p* < 0,001), schwere unerwünschte Arzneimittelwirkungen jedoch seltener (23,50 % vs. 25,40 %; *p* < 0,05; Abb. 2).

In CANVAS (2017) wurden kardiovaskulärer Tod, Myokardinfarkt und Schlaganfall als zusammengesetzter primärer Endpunkt unter Canagliflozin bei 10.142 Patienten versus Placebo analysiert [7]. Nur für die Population der Teilstudie CANVAS‑R (5812 Patienten) werden explizite Daten zu Hospitalisationen wegen HI berichtet. Von diesen Patienten mit Diabetes mellitus Typ 2 hatten 14,4 % anamnestisch eine HI, bei 20,1 % lag die eGFR zwischen 30 und 60 ml/min/1,73 m^2^. Der kombinierte sekundäre Endpunkt aus kardiovaskulärem Tod und HI-bedingten Hospitalisationen unterschied sich nur in Letzteren signifikant zugunsten von Canagliflozin (HR: 0,72; 95 %-KI: 0,55–0,94; *p* = 0,0148). Die Ereignisrate, bezogen auf 1000 Patientenjahre, betrug 15,85 unter Canagliflozin und 21,91 unter Placebo (NNT wegen fehlender absoluter Zahlen unberechenbar). Körpergewicht (−3,9 vs. −1,6 kg) und systolischer Blutdruck (−5,2 vs. −2,5 mm Hg) nahmen unter Canagliflozin signifikant stärker ab als unter Placebo. Unter Canagliflozin waren die unerwünschten Arzneimittelwirkungen osmotische Diurese/Volumendepletion 1,4fach und die Zahl peripherer Amputationen fast 2fach signifikant erhöht. Die Studienabbruchrate lag unter Canagliflozin bei 29,20 % im Vergleich zu 29,90 % unter Placebo. In dieser Untersuchung konnte bei auch hier niedriger Ereignisrate der in EMPA-REG OUTCOME gesehene positive Effekt auf HI-bedingte Hospitalisationen reproduziert werden (Abb. 2).

DECLARE-TIMI 58 (2018) ist mit über 17.160 Teilnehmern die größte der 3 randomisierten Studien [8]. Eine HI war bei 9,90 % der Teilnehmer in der Verum- und bei 10,20 % in der Placebogruppe vorbekannt, bei 7,00 % von ihnen lag die eGFR bei der Randomisierung unter 60 ml/min/1,73 m^2^ (eigentlich Ausschlusskriterium). HI-bedingte Hospitalisationen als Bestandteil des primären Effektivitätsendpunkts waren unter Dapagliflozin seltener als unter Placebo (2,47 vs. 3,33 %; absolute Risikoreduktion: 0,86; NNT: 117; HR: 0,73 [95 %-KI: 0,61–0,88]; *p* = 0,005 für die Kombination aus HI-bedingter Hospitalisation und kardiovaskulärem Tod, nur ersteres unterschiedlich). Nach 48 Monaten zeigten sich unter Dapagliflozin eine stärkere Gewichtsabnahme (−4,00 vs. −2,00 kg) und Reduktion des systolischen Blutdrucks (−3,00 vs. 0,00 mm Hg) als unter Placebo. Der kombinierte renale Endpunkt (≥40 % eGFR-Abnahme auf <60 ml/min/1,73 m^2^, terminale Niereninsuffizienz oder Tod renaler Ursache) trat unter dem Verum seltener auf als unter Placebo (4,30 vs. 5,60 %; HR: 0,76 [95 %-KI: 0,67–0,87]; *p:* nicht angegeben). Ketoazidosen waren häufiger (0,30 % vs. 0,10 %; HR: 2,18 [95 %-KI: 1,1–4,3]; *p* = 0,02), ebenso genitale Infektionen (0,90 % vs. 0,10 %, HR: 8,36 [95 %-KI: 4,19–16,68]; *p* < 0,001). Die Abbrecherquote lag aber unter Dapagliflozin niedriger als unter Placebo (21,10 % vs. 25,10 %). Insgesamt zeigte die Studie einen risikomindernden Effekt von Dapagliflozin auf HI-bedingte Hospitalisationen bei hoher NNT (Abb. 2).

Eine kleinere randomisierte Studie, die Dapagliflozin versus Placebo an 922 Diabetes-mellitus-Typ-2-Patienten untersuchte, wurde 2015 publiziert [9]. Bei kardiovaskulär Vorerkrankten mit einer eGFR von 60 ml/min/1,73 m^2^ oder darüber wurde HI lediglich als Sicherheitsendpunkt untersucht. Schwere unerwünschte Arzneimittelwirkungen in Bezug auf HI waren dabei nicht unterschiedlich, jedoch gab es mehr unspezifische kardiale unerwünschte Arzneimittelwirkungen in der Dapagliflozin- als in der Placebogruppe (15,40 vs. 12,50 %), entsprechend einer Anzahl von Behandlungen pro schädigendes Ereignis von 34,48. Hypotension/Dehydratation oder Hypovolämie trat unter Dapagliflozin deutlich häufiger auf als unter Placebo (2,80 vs. 0,40 %), entsprechend einer Anzahl von Behandlungen pro schädigendes Ereignis von 42. Die Studienabbruchrate war gering (Dapagliflozin: 12,39 %; Placebo: 12,55 %). Nach 24 Wochen kam es unter dem Verum zu einer stärkeren Gewichtsabnahme (−2,56 % vs. −0,30 %; *p* < 0,0001) und Senkung des systolischen Blutdrucks (−2,0 mm Hg; −2,99 % vs. −1,03 %;* p* < 0,05) als unter Placebo (Abb. 2).

### Retrospektive Beobachtungsstudien und Post-hoc-Analysen

2 retrospektive Beobachtungsstudien [10, 11] verglichen, basierend auf Gruppen mit vergleichbarem Risiko, verschiedene SGLT2i (hauptsächlich Dapagliflozin und Canagliflozin) mit anderen oralen Antidiabetika. Insgesamt wurden die Verläufe von 428.014 Patienten u. a. mit dem Endpunkt HI-bedingte Hospitalisation untersucht, dieser ereignete sich in beiden Studien unter dem jeweiligen SGLT2i signifikant seltener als unter anderen oralen Antidiabetika. In der größeren Studie mit 309.056 Patienten (prozentuale Expositionszeit: 52,70 % für Canagliflozin, 42,00 % für Dapagliflozin, 5,50 % für Empagliflozin) kam es unter SGLT2i seltener zu einer HI-bedingten Hospitalisation (0,237 vs. 0,384 %; absolute Risikoreduktion: 0,147; NNT: 681; HR: 0,61 [95 %-KI: 0,51–0,73]; *p* < 0,001; [10]). In der zweiten Studie (90,2 % Dapagliflozin) lag die Rate der HI-bedingten Hospitalisationen unter SGLT2i ebenfalls niedriger als unter Hemmstoffen der Dipeptidylpeptidase 4 (DPP-4-Hemmer; 0,83 vs. 1,13 Ereignisse/100 Personenjahre; absolute Risikoreduktion/NNT: nicht berechenbar; HR: 0,66 [95 %-KI: 0,58–0,75]; *p* < 0,001; [11]). In beiden Untersuchungen war die Zahl der Hospitalisationen wegen HI sehr niedrig (0,31 bzw. 0,86 %).

Eine Post-hoc-Analyse einer randomisierten Studie mit Canagliflozin versus Placebo mit 666 Teilnehmern untersuchte die Entwicklung von Biomarkern unter SGLT2i-Therapie [12]. U. a. wurde der HI-spezifische Marker NT-proBNP (N-terminales Propeptid „brain natriuretic peptide“) analysiert, der unter erhöhter myokardialer Wandspannung ansteigt. In beiden Behandlungsgruppen kam es nach 2 Jahren zu einem Anstieg des NTproBNP-Medians; dieser war unter Canagliflozin statistisch signifikant geringer als unter Placebo (2,4 vs. 12,5 pg/ml; *p* < 0,01). Dabei lagen aber die absoluten NTproBNP-Werte im Median unter Canagliflozin höher als unter Placebo (47,4 pg/ml vs. 43,4 pg/ml), beide im unteren Normbereich [2].

### Metaanalysen

Die untersuchten Metaanalysen sind von den 3 oben genannten großen randomisierten Studien dominiert. 9 von 10 ergaben eine positive Auswirkung von SGLT2i auf HI. Diejenige Metaanalyse mit vorbestehender HI als Einschlusskriterium erbrachte kein signifikantes Ergebnis zugunsten von Dapagliflozin [13]. 3 andere Metaanalysen zeigten einen positiven Effekt von Empagliflozin, nicht aber anderer SGLT2i in Bezug auf HI, die hier über Hospitalisation wegen HI hinausgehend definiert wurde [14–16]. In einer weiteren Analyse wurden nur Patienten mit einer eGFR von weniger als 60 ml/min/1,73 m^2^ untersucht; die eGFR lag in den einbezogenen Studien im Bereich von im Mittel 38,00 bis im Mittel 53,50 ml/min/1,73 m^2^ [17]. Die Untersuchung des HI-Endpunkts (HI-bedingte Hospitalisation oder „fatale HI“) erfolgte in Bezug auf Empagliflozin, Canagliflozin und Dapagliflozin. Die Rate an HI-Ereignissen war mit 3,79 % die höchste aller hier präsentierten Studien, und sie war unter SGLT2i niedriger als unter der jeweiligen Vergleichstherapie aus Placebo oder aktivem Komparator (absolute Risikoreduktion: 2,43 %, NNT: 42, HR: 0,61 [95 %-KI: 0,48–0,78]; *p*: nicht angegeben). In weiteren Studien zeigten sich positive Auswirkungen der SGLT2i (dominierend: Empagliflozin, zumeist gegen Placebo getestet) auf HI-Ereignisse mit NNT-Werten von 72, 84, 91 und 625 [18–21]. Und schließlich erschien eine Metaanalyse der 3 großen randomisierte Studien, die das gesamte Patientenkollektiv, aufgeteilt nach eGFR-Subgruppen, nachuntersuchte [22]. Die Patienten mit einer eGFR von weniger als 60 ml/min/1,73 m^2^ profitierten am stärksten in Bezug auf die Reduktion von HI-bedingten Hospitalisationen (relativ: 40 %, absolute Risikoreduktion: nicht verfügbar). Lag die eGFR zwischen 60 und 90 ml/min/1,73 m^2^, betrug die relative Risikoreduktion 31 %. In der Gruppe mit einer eGFR von 90 ml/min/1,73 m^2^ oder darüber ließ sich kein signifikanter Effekt darstellen.

## Diskussion

**Figure 3:**
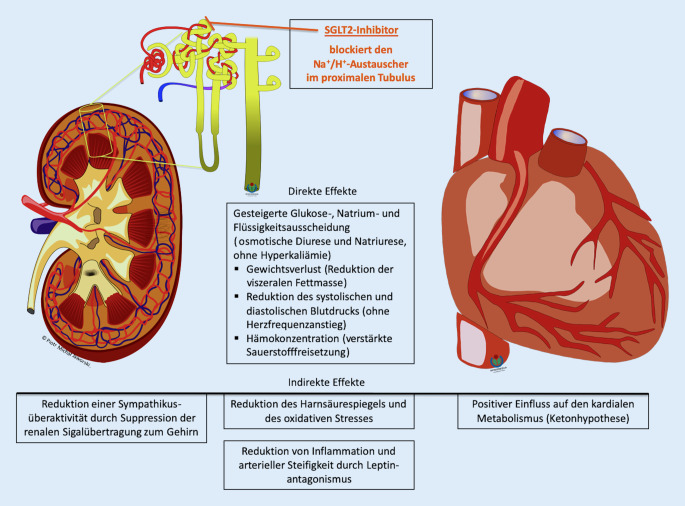

Seit Publikation der EMPA-REG-OUTCOME-Studie ist es zu einem starken Interesse an dem beobachteten „Anti-Herzinsuffizienz-Effekt“ gekommen, als dessen Grundlage eine Vielzahl pharmakologischer Effekte der SGLT2i vermutet werden ([23]; Abb. 3). Diese Wirkungen hängen teilweise direkt mit deren Hemmung der Natrium-Glukose-Reabsorption im proximalen Tubulus zusammen, die zu einer Natriurese und einer osmotischen Diurese durch Glukosurie führt. Dadurch entsteht ein diuretischer Effekt, der zu Volumenreduktion, Gewichtsabnahme und Blutdrucksenkung führt. Die Mechanismen, durch welche die vielfach belegte nephroprotektive Wirkung der SGLT2i zustande kommt, werden als „wahrscheinlich multifaktoriell“ beschrieben, mit einer vermutlich wichtigen Rolle renovaskulärer Effekte. Eine Aktivierung der tubuloglomerulären Rückkopplung mit Senkung des intraglomerulären Drucks und Reduktion der Hyperfiltration wurde experimentell unter Empagliflozin beobachtet [24, 25]. Darüber hinaus werden verminderter oxidativer Stress und Verbesserung des kardialen Metabolismus diskutiert. Eine Hypothese besagt, dass die osmotische Diurese unter SGLT2i eine Volumenreduktion im Interstitium unter Aufrechterhaltung des notwendigen intravaskulären Volumens bewirkt. Eine HI könnte hierdurch günstiger beeinflusst werden als unter einer Diuretikatherapie, die bevorzugt das intravaskuläre Volumen reduziert [26]. In einer Mediationsanalyse (statistisches Verfahren, das Kausalität und zeitliche Abfolge zwischen verschiedenen Variablen darstellen soll) von EMPA-REG OUTCOME wurde der Anstieg von Hämatokrit und Hämoglobin als wichtigster Mediator der kardiovaskulären Risikoreduktion von Empagliflozin gegenüber Placebo ermittelt [27], was für den positiven Effekt einer Reduktion des Plasmavolumens spricht. Daten der DAPA-HF(Dapagliflozin And Prevention of Adverse Outcomes in Heart Failure)-Studie [28], die im November 2019 (und damit erst nach der Datenbankrecherche für diese Übersicht) publiziert wurde, ist jedoch zu entnehmen, dass die Behandlung mit Dapagliflozin zu keiner wesentlichen Änderung des Diuretikaregimes geführt hat. Demnach konnte ein „diuretikasparender Effekt“ von SGLT2i in der klinischen Anwendung bisher nicht belegt werden.

In allen referierten Studien waren signifikante Körpergewichts- und Blutdruckreduktionen unter SGLT2i zu verzeichnen, wobei sich diese Abnahme beim Gewicht zwischen −2,9 und−4 kg, beim systolischen Blutdruck zwischen −2,0 und −5,2 mm Hg bewegte. Dieser geringe Unterschied allein reicht zur Erklärung des verringerten HI-Risikos vermutlich nicht aus. In anderen Studien war eine deutlich höhere Blutdrucksenkung zur Senkung des HI-Risikos notwendig. In der SPRINT(Systolic Blood Pressure Intervention Trial)-Studie mit Hypertonikern waren dies −20 mm Hg in der Gruppe mit intensivierter Therapie versus −8 mm Hg unter Standardtherapie [29]. Um einen HI-protektiven Effekt durch Gewichtsabnahme bzw. Reduktion des systolischen Blutdrucks nachzuweisen, wären Subgruppenanalysen notwendig, die bisher nicht publiziert sind.

Die Datenlage zu Nierenfunktion und HI-Protektion ist besser: Mehrere Arbeiten zeigen Hinweise auf eine ausgeprägtere HI-Risiko-Minderung bei stärker eingeschränkter Ausgangsnierenfunktion. Bei bekannter Interdependenz dieser beiden Organsysteme ist die Niereninsuffizienz eine gravierende Komplikation bei fortgeschrittener HI. Demnach könnte eine SGLT2i-Therapie über Nephroprotektion zu besserer Volumenhomöostase und zu geringerer neurohumoraler Aktivierung führen [30]. Bei einer manifesten HI sind diese beiden Regelkreise gestört [2]. Weiterhin könnte sich die Verhinderung/Progressionshemmung einer diabetischen Kardiomyopathie [1] durch eine verbesserte glykämische Kontrolle günstig auswirken. Allerdings war die Rate manifester ischämischer Ereignisse in EMPA-REG OUTCOME nicht signifikant unterschiedlich.

Die oben genannte DAPA-HF-Studie konnte einen symptomatik- und prognoseverbessernden Effekt von Dapagliflozin in einem Hochrisikokollektiv (HI-Patienten mit LVEF [linksventrikuläre Ejektionsfraktion] <40 %) unabhängig von einer Diabeteserkrankung nachweisen (NNT = 21). Damit kann ein HI-spezifischer Effekt der SGLT2i als erwiesen gelten, ohne dass mit dieser Studie über die zugrunde liegenden Mechanismen mehr bekannt geworden wäre.

Weitere, aktuell laufende Studien wie EMPEROR (‑Reduced [Empagliflozin Outcome Trial in Patients With Chronic Heart Failure With Reduced Ejection Fraction], EudraCT-Nr. 2016-002280-34, und -Preserved [Empagliflozin Outcome Trial in Patients With Chronic Heart Failure With Preserved Ejection Fraction], Nr. 2016-002278-11), DELIVER (Dapagliflozin Evaluation to Improve the Lives of Patients With Preserved Ejection Fraction Heart Failure; EudraCT-Nr. 2018-000802-46) und SOLOIST-WHF (Effect of Sotagliflozin on Cardiovascular Events in Patients With Type 2 Diabetes Post Worsening Heart Failure; EudraCT-Nr. 2017-003510-16) bei Patienten mit diabetischer und nichtdiabetischer HI und einem primären HI-Endpunkt werden vielleicht auch Aussagen darüber zulassen, ob die Wirkung der SGLT2i sich je nach Typ der HI – mit erhaltener oder reduzierter LVEF – unterscheidet.

## Fazit für die Praxis

- Die Häufigkeit Herzinsuffizienz(HI)-bedingter Hospitalisationen konnte durch SGLT2(„sodium glucose-linked transporter 2“)-Inhibitoren (SGLT2i) in Studien gesenkt werden.
- Ein Klasseneffekt wird vermutet, es liegen aber praktisch nur zu Empagliflozin, Canagliflozin und Dapagliflozin Daten vor. Die beobachteten hohen NNT („number needed to treat“) beruhen am ehesten auf geringen HI-Ereignis-Raten in der untersuchten Population.
- Subgruppenanalysen an Patienten mit bereits manifester HI vor Studieneinschluss ließen keine Unterschiede im Vergleich zu den anderen Studienteilnehmern erkennen, sodass vor Publikation von DAPA-HF ein HI-spezifischer Effekt unsicher erschien.
- Der HI-präventive Effekt der SGLT2i ist nach gegenwärtigem Kenntnisstand am ehesten durch einen nephroprotektiven Effekt zu erklären, da Patienten mit reduzierter Nierenfunktion in Subgruppenanalysen deutlich stärker von der Therapie profitierten als diejenigen ohne Niereninsuffizienz.
- Möglicherweise bedingt die unter SGLT2i-Therapie nachgewiesene Besserung der glomerulären Hyperfiltration eine positive Modulation der bei HI in schädlicher Weise überaktivierten renalen Hormonsysteme.
